# Measuring Comfort Behaviours in Laying Hens Using Deep-Learning Tools

**DOI:** 10.3390/ani13010033

**Published:** 2022-12-21

**Authors:** Marco Sozzi, Giulio Pillan, Claudia Ciarelli, Francesco Marinello, Fabrizio Pirrone, Francesco Bordignon, Alessandro Bordignon, Gerolamo Xiccato, Angela Trocino

**Affiliations:** 1 Department of Land, Environment, Agriculture and Forestry (TeSAF), University of Padova, Viale dell’Università 16, 35020 Padova, Italy; 2 Department of Comparative Biomedicine and Food Science (BCA), University of Padova, Viale dell’Università 16, 35020 Padova, Italy; 3 Department of Agronomy, Food, Natural resources, Animal and Environment (DAFNAE), University of Padova, Viale dell’Università 16, 35020 Padova, Italy

**Keywords:** cage-free systems, dust bathing, machine learning, YOLO, image analyses

## Abstract

**Simple Summary:**

Precision livestock farming (PLF) techniques facilitate automated, continuous, and real-time monitoring of animal behaviour and physiological responses. They also have the potential to improve animal welfare by providing a continuous picture of welfare states, thus enabling fast actions that benefit the flock. Using a PLF technique based on images, the present study aimed to test a machine learning tool for measuring the number of hens on the ground and identifying the number of dust-bathing hens in an experimental aviary—a complex environment—by comparing the performance of two machine learning (YOLO, You Only Look Once) models. The results of the study revealed that the two models had a similar performance; however, while PLF was successful in evaluating the distribution of hens on the floor and predicting undesired events, such as smothering due to overcrowding, it failed to identify the occurrence of comfort behaviours, such as dust bathing, which are part of the evaluation of hen welfare.

**Abstract:**

Image analysis using machine learning (ML) algorithms could provide a measure of animal welfare by measuring comfort behaviours and undesired behaviours. Using a PLF technique based on images, the present study aimed to test a machine learning tool for measuring the number of hens on the ground and identifying the number of dust-bathing hens in an experimental aviary. In addition, two YOLO (You Only Look Once) models were compared. YOLOv4-tiny needed about 4.26 h to train for 6000 epochs, compared to about 23.2 h for the full models of YOLOv4. In validation, the performance of the two models in terms of precision, recall, harmonic mean of precision and recall, and mean average precision (mAP) did not differ, while the value of frame per second was lower in YOLOv4 compared to the tiny version (31.35 vs. 208.5). The mAP stands at about 94% for the classification of hens on the floor, while the classification of dust-bathing hens was poor (28.2% in the YOLOv4-tiny compared to 31.6% in YOLOv4). In conclusion, ML successfully identified laying hens on the floor, whereas other PLF tools must be tested for the classification of dust-bathing hens.

## 1. Introduction

Precision livestock farming (PLF) techniques facilitate the automated, continuous, and real-time monitoring of animal behaviour and physiological responses both at an individual level and at a group level, depending on the farmed species [1]. Under the conditions of poultry production, PLF tools can greatly assist farmers in taking the correct action [2,3,4,5] to guarantee the health and welfare of thousands of animals when environmental conditions are unfavourable [3], illness spreads [6,7], or abnormal behaviours challenge bird welfare and survival [8,9]. In poultry, these systems have received increased attention on a global scale starting from 2020. Commercial applications include several sensors for the continuous measuring of environmental conditions (temperature, ambient dust, relative humidity, vibration, ammonia concentration, carbon dioxide concentrations), including a camera system (Fancom BV) to monitor the distribution and activity of chickens and a sensor for detecting shell thickness and cracked eggs [10].

PLF tools based on images recorded by cameras have been used to obtain information about farm/animal temperature levels, the quality of activity, and animal behaviours, as well as performance [11]. Some of these studies used images to evaluate changes in bird behaviour following changes in environmental conditions or stocking density, even at the nest level in the case of laying hens [12]. The commercially available eYeNamic™ (Fancom BV, The Netherlands) uses images of animal distribution in broiler chicken farms to alert farmers about malfunctioning environmental control and feeding systems and has been found to be effective in identifying 95% of the problems occurring in a production cycle [9]. As for laying hens, since the housing environment is more complex compared to broiler chickens, the development of PLF tools needs to be specific to housing.

In Europe, more than 44% of hens are kept in enriched cages [13], which are intended to provide additional space and resources for satisfying hen behavioural needs; however, conventional cage-free barn systems, including aviaries and free-range and organic systems with outdoor access, are becoming popular and are expected to replace cages soon. In fact, the European Resolution P9_TA(2021)0295 calls for a phasing out of cages by 2027 and the full implementation of cage-free systems, while the European Green Deal and farm-to-fork strategies require more sustainable animal production systems. However, experience in the field and the literature have identified weaknesses in addition to strengths of cage-free systems for the production and welfare of hens [14,15]. As such, we require further insights regarding the identification of on-farm solutions for the control of challenging situations, as well as for farmers’ actions, to prevent major welfare and health issues, where PLF tools could provide great help.

Hens are often synchronous in their behaviours (e.g., movement, dust bathing, laying), which can lead to overcrowding in specific parts of the aviary and provoke unusual behaviours, such as flock piling, i.e., dense clustering of hens mainly along walls and in corners which can result in smothering and high losses [16,17]. In cage-free systems, the possibility of monitoring hen crowding is also crucial for other behaviours, where the expression of some comfort behaviours, such as dust bathing [18,19], may be affected by the available space on the ground [16,20].

Image analysis is a suitable methodology that uses cameras to estimate a number of objects (e.g., number of hens). Among the different image analysis techniques, machine learning (ML) algorithms have proven to be the most effective for object detection. The most-used ML methods in agriculture include dimension reduction, regressions, clustering, k-means, Bayesian models, k-nearest neighbours, decision trees, support vector machines, and artificial neural networks [21,22]. The foundation of artificial neural networks is a network of interconnected nodes that are arranged in a certain topology. When it comes to deep neural networks, there are numerous layers in addition to the single layer of the perceptron [22]. Deep neural networks are commonly referred to as deep learning. The latter has a higher performance and surpasses other ML strategies in image processing according to a meta-analysis of ML by Kamilaris and Prenafeta-Boldú [21]. Convolutional neural networks (CNN) are one of the most significant deep learning models used for image interpretation and computer vision [21]. They can be used to analyse, combine, and extract colour, geometric, and textural data from images. The two primary frameworks on which object-identification models are based are as follows. The first is based on region proposals and classifies each proposal into several object categories; the second treats object detection as a regression or classification problem [23]. The output of object detection is typically bounding boxes over the image, but some models produce semantic segmentation as the result. R-CNN, Faster R-CNN, and Mask R-CNN are examples of region-proposed object-detection algorithms. At the same time, You Only Look Once (YOLO) [24,25,26] and single shot detector (SSD) are examples of regression/classification-based models [27].

Thus, using a PLF technique based on images, the present study aimed to test an ML tool to measure the number of hens on the ground and identify the number of dust-bathing hens in an experimental aviary. In addition, the performance of two YOLO models was compared, with the aim of developing an alert tool for abnormal crowding and a monitoring tool for comfort behaviours and welfare indicators.

## 2. Materials and Methods

### 2.1. Animals and Housing

A total of 1800 Lohmann Brown-Classic hens (Lohmann Tierzucht GmbH, Cuxhaven, Germany), aged 17 weeks, were housed at the experimental farm “Lucio Toniolo” of the University of Padova (Legnaro, Padova, Italy) and were randomly divided into 8 pens in an experimental aviary system (225 hens per pen; 9 hens/m^2^ stocking density), where they were monitored until 45 weeks of age within a specific research project [28].

The experimental farm building was equipped with a cooling system, forced ventilation, radiant heating, and controlled lighting systems. The experimental aviary specifically set up in the farm consisted of two tiers equipped with collective nests (1 nest per 60 hens) that were closed by red plastic curtains, and a third level with only perches and feeders. The two tiers also included perches, nipple drinkers, and automatic feeding systems. The whole aviary system was 2.50 m wide × 19.52 m long × 2.24 m high, and two corridors per side were available, each 1.70 m wide. Thus, free ground space was 5.90 m wide × 19.52 m long. Litter was based on manure of hens. The aviary was divided into 8 pens each with a length of 2.44 m.

### 2.2. Video Recordings and Test Sets

The aviary was equipped with a real-time video recording system, which used 48 cameras (infrared mini-dome bullet 4 mp; resolution 1080 p) (HAC-HDW1220MP, Zhejiang Dahua Technology Co., Ltd., Hangzhou, China) and 2 full HD video recorders (NVR2116HS-4KS2, Zhejiang Dahua Technology Co., Ltd., Hangzhou, China). The cameras were located to record hens on the ground, hens on the three levels of the aviary, and hens in the nests. One camera per pen was used to record the animals on the floor. Cameras were hung at a height of 2 m with a dome angle of 180° in the middle of each pen. The following settings were used: video recording frame rate at 30 fps, backlight compensation as digital wide dynamic range, auto white balance, and video compression as H.265.

Once per week (Saturday) during the trial from 38 to 45 weeks of age, the behaviour of hens was video recorded from 5:30 until 19:30 during the light hours when the animals were active. Of all video-recorded data, 112 hours of video recordings of the hens on the ground floor were used. The software “Free Video to JPG Converter (v. 5.0.101)” was used to extract 1 frame per second throughout the 112 hours, totalling 403,200 extracted frames. A limited number of images were selected from the whole dataset to avoid autocorrelation between frames and achieve a significant number of labels for hens on the floor and dust-bathing hens. In addition, a sufficient but not excessive number of images makes this methodology applicable for commercial applications by farmers and consultants. Then, 1150 images were randomly selected for the purposes of the present study. A total of 1100 images were used as the training set; the remaining 50 were used as the validation set.

### 2.3. Set up of the Object-Detection Algorithm

YOLO addresses object detection as a single regression problem, avoiding the region proposal, classification, and duplicating elimination pipeline. In recent years, different versions of YOLO have been proposed (YOLO 9000, YOLOv2-v3-v4-v5, Fast YOLO, versions tiny), but we used two versions of YOLO: YOLOv4-tiny and YOLOv4 [24,25,26]. They are both quick convolutional neural networks that can classify images based on bounding box labelling [29,30]. The two versions of YOLOv4 were selected since they provide a good trade-off between accuracy and digital effort [31].

YOLOv4 is more precise than YOLOv4-tiny [32] because it comprises a higher number of convolutional layers (53 vs. 36). YOLOv4-tiny is expected to be faster but less accurate in its predictions given its reduced number of convolutional layers.

Training and data analysis of YOLO were carried out using the Python programming language. Darknet framework, an open-source neural network framework written in C and CUDA, was installed on a virtual machine on Google Colaboratory. YOLOv4 and YOLOv4-tiny are based on the CSPDarknet53, which includes cross-stage partial connections to the Darknet framework [33]. The cross-stage partial connections divide the input features into two groups: one group is processed by the convolutional layer, while the second sidesteps the convolutional layers and is included in the input for the following layer [34].

**Figure 1:**
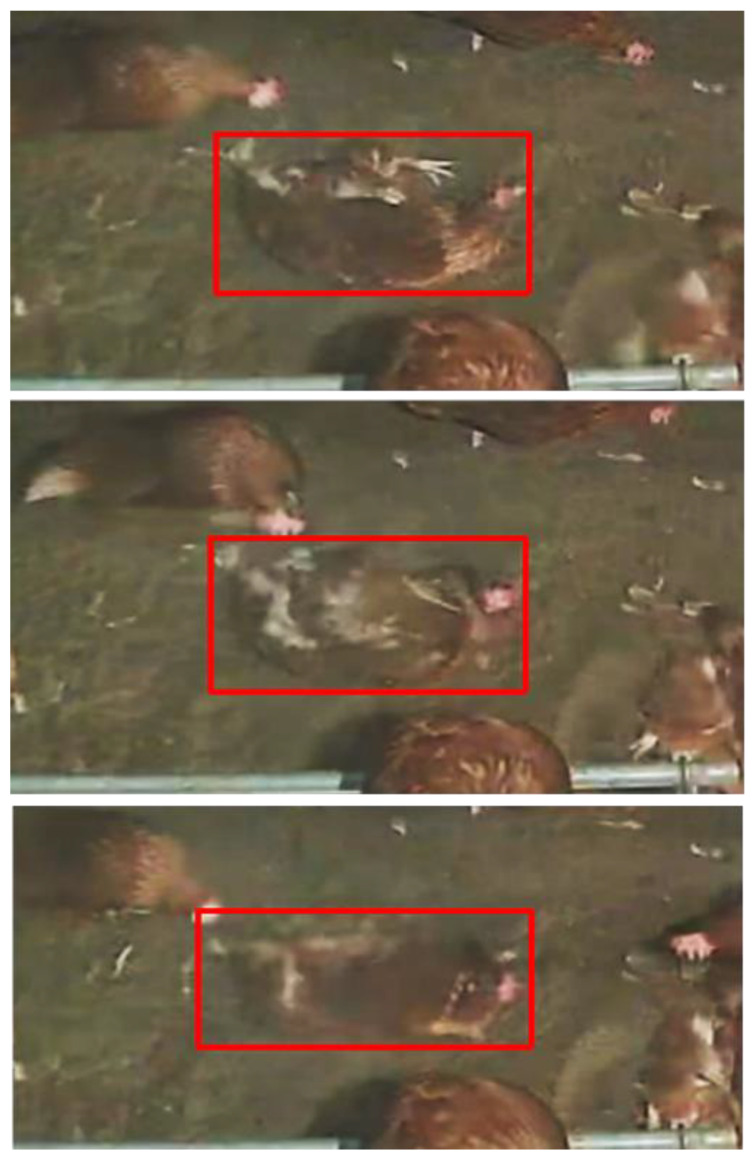
A dust-bathing hen (in red boxes) in three consecutive frames, as labelled for the training of the model.

From the whole dataset (403,200 frames), we selected a sample of 1150 images for training. The choice of the sample size was based on the slow movement of animals, for which the images acquired every second slightly differed from each other. Before training, all 1150 frames were labelled by bounding boxes distinguishing between “dust-bathing” hens (hens rotating their body in the litter on the ground) and hens on the “ground” (all the other hens, excluding those climbing towards or landing from the aviary) (Figure 1). The images collected from the dataset mentioned above were manually labelled, drawing bounding boxes on each bunch in the image using YOLO_label V2 project [35]. YOLO_label allows us to create annotations (labels) for the object-detection algorithm using the YOLO_label format, which consists of five columns for each object (object-class, x, y, width, and height).

### 2.4. Training and Validation

**Figure 2:**
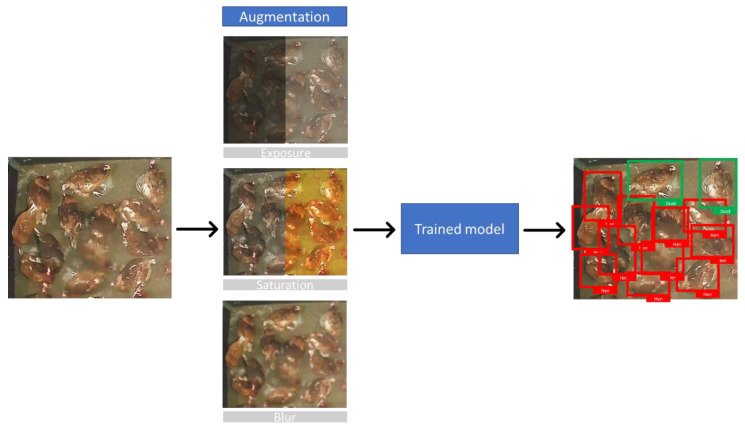
Augmentation and classification scheme for laying hens on the floor (red boxes) and dust-bathing hens (green boxes).

Using the specified dataset, the YOLOv4 and YOLOv4-tiny models were trained individually. The pre-trained weights provided by the YOLO developers were used in the training operations [33,36]. Each model performed a 6000-epoch training phase during which detailed calibration of the training hyperparameters was carried out. An epoch is defined as the duration required for one training step. For the best training results, hyperparameters can be modified in the YOLO configuration file. The first section of a configuration file lists the batch size (number of photos utilized each epoch) as well as the dimensions of the resampled images used for training (width and height). An image batch size of 64 images with a pixel size of 608 × 608 was used for training and detection. Online data augmentation was activated in the configuration file for YOLOv4 full models. Data augmentation techniques were introduced in the training process for unobserved data, which were obtained from combinations and modification of the input dataset. In YOLO models, data augmentation randomly applies graphics modification to the input images [37]. For each training period, a different approach to online data augmentation can be applied. In the current training, images were augmented in terms of saturation and exposure, using a coefficient of 1.5. Hue value was randomly augmented with a coefficient of 0.05 (Figure 2). Additionally, random blur and mosaic effects were applied to the input photos. Because of the mosaic created by combining pieces of many photographs to produce a new tiled image, blur increased the fuzziness of the input images. The starting learning rate and its scheduler were chosen in the configuration file. The learning rate controls the adaptation of the models according to the error estimation in each training epoch. The initial learning rate was 0.002 for YOLOv4 and YOLOv4-tiny training. A total of 384,000 augmented images were used based on the batch size (64 images) and the number of epochs (6000).

All models were trained using the stochastic gradient descent with warm re-starts (SGDR) scheduler [38]. Following a cosine cycle, the SGDR reduces the learning rate from its initial value all the way down to zero. The user specifies the number of epochs for each cycle. In order to increase the number of epochs for a cycle during training, the cycle may be multiplied by a coefficient. The initial SGDR cycle used in this study had a multiplier coefficient of 2- and it lasted for 1000 epochs.

The results of the training were evaluated for precision, recall, and F1-score, as well as mean average precision (mAP) and frame per second (FPS). The F-1 score, precision, and recall were calculated according to Equation (1): Performance metric used to evaluate models’ performances.

(1) $$F1-\mathrm{score}=2\times \frac{\mathrm{Precision}\times \mathrm{Recall}}{\mathrm{Precision}+\mathrm{Recall}\;}\;\begin{array}{c} \mathrm{while}\\ \mathrm{Precision}=\frac{\mathrm{True}\;\mathrm{Positive}}{\mathrm{True}\;\mathrm{Positive}+\mathrm{False}\;\mathrm{Negative}}\;\mathrm{and}\;\mathrm{Recall}=\frac{\mathrm{True}\;\mathrm{Positive}}{\mathrm{True}\;\mathrm{Positive}+\mathrm{False}\;\mathrm{Positive}} \end{array}$$

The F-1 score represents the harmonic mean of precision and recall, and it was introduced by Dice [39] and Sørensen [40]. Intersection over Union (IoU) [41] and mAP were used as performance metrics. The IoU measures the grade of overlap between the predicted and the labelled bounding boxes. The mAP summarizes the average detection precision and represents the area under the precision–recall curve at a defined value of IoU. The mAP@50 represents the area under the precision–recall curve with a grade of overlapping bounding boxes of 50%. Performance metrics calculation was evaluated on the validation datasets by running the object-detection algorithm obtained by the training.

Frame per second (FPS) expresses the speed achieved by the neural network, that is, the number of images per second that the network can process. Colab provides a virtual machine with the Ubuntu operating system (Canonical Ltd., London, UK), equipped with an Intel Xeon (Intel Corporation, Santa Clara, CA, USA) processor with two cores at 2.3 GHz. In Colab, 25 GB of random-access memory (RAM) is available. Training and detection tasks were performed taking advantage of a Tesla P100 GPU (NVIDIA, Santa Clara, CA, USA) with CUDA parallel computing platform version 10.1 and 16 GB of dedicated RAM.

## 3. Results

**Figure 3:**
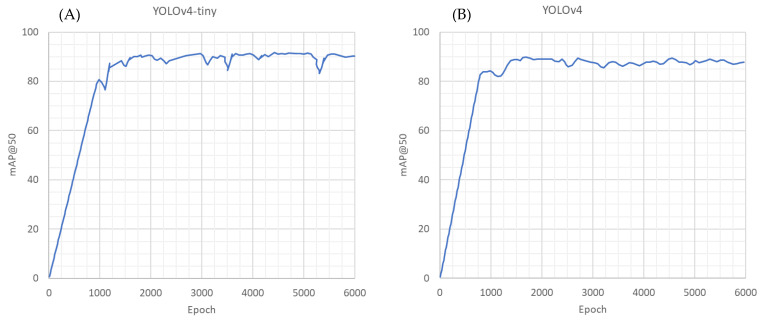
Area under the precision–recall curve with a grade of overlapping bounding boxes of 50% (mAP@50) of YOLOv4-tiny training (**A**) and YOLOv4 training (**B**).

For each YOLO model, the training process (Figure 3) was conducted using the indicated configuration. The model performance on the test dataset was automatically estimated during training. Every 100 epochs, the mAP@50 was calculated, with the last calculation occurring at 6000 epochs. Table 1 reports the last and the best mAP@50 achieved during training, along with the total time spent for 6000 training epochs. YOLOv4-tiny needed about 4.26 hours to train for 6000 epochs (final mAP@50 of 90.3%, best mAP@50 91.7%), compared to about 23.2 hours for the full models of YOLOv4 (final mAP@50 of 87.8%, best mAP@50 90.0%).

### Validation of the External Dataset

**Figure 4:**
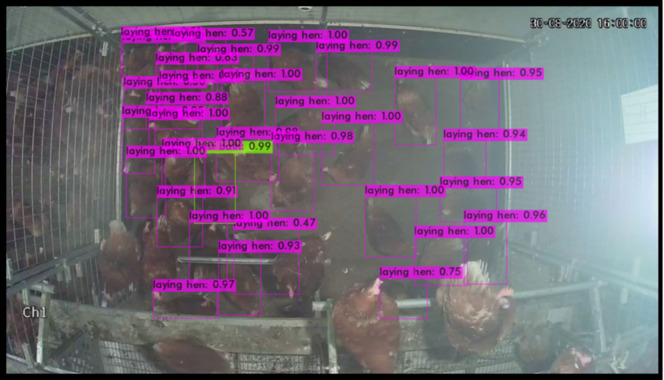
Object-detection classification performed on an image of the validation set using YOLOv4-tiny. Purple boxes identify images as laying hen with the corresponding confidence interval. Green boxes identify images as dust-bathing hens with the corresponding confidence interval.

**Figure 5:**
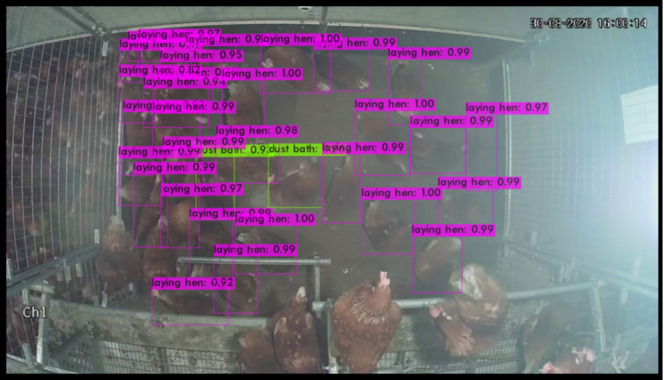
Object-detection classification performed on an image of the validation set using YOLOv4. Purple boxes identify images as laying hen with the corresponding confidence interval. Green boxes identify images as dust-bathing hens with the corresponding confidence interval.

Validation was carried out by running the object-detection algorithm on the validation set (50 images randomly selected), which was not used for the training (Figure 4 and Figure 5). Performance of the two models regarding IoU, precision, recall, F1-score, and mAP did not substantially differ, while FPS was largely lower in the case of YOLOv4 compared to the tiny version (31.35 vs. 208.5) (Table 2). Mean average precision stood at 61.4% and 62.9%, while large differences were recorded in mAP for the classification of hens on the floor, with high values around 94%, and a poor classification of dust-bathing hens, ranging from 28.2% in the YOLOv4-tiny compared to 31.6% in the YOLOv4.

## 4. Discussion

Previously, PLF tools based on images have been successfully used to detect foot problems in broiler chickens [8], to classify sick and healthy birds based on their body posture [6], and to classify species-specific behaviours with an overall success rate of 97% and 70% in calibration and validation, respectively [42].

Pu et al. [43] proposed an automatic CNN to classify the behaviours of broiler chickens based on images acquired by a depth camera under three stocking-density conditions (high, medium, and low crowding in a poultry farm). The latter CNN architecture reached an accuracy of 99.17% in the classification of flock behaviours.

In poultry breeders, six behaviours were classified under low- and high-stocking density in a combined wire cage system with two pens and using the deep-learning YOLOv3 algorithm [44]. The model always identified several behaviours with different but always high degrees of accuracy, i.e., mean precision rate of 94.72% for mating; 94.57% for standing; 93.10% for feeding; 92.02% for spreading; 88.67% for fighting; and 86.88% for drinking. The accuracy of the model was lower in the high-density cages compared to the low-density cages due to shielding among the birds. Based on these results, the same authors succeeded in evaluating animal welfare on different observation days based on the frequencies of mating events and abnormal behaviours, where the latter were related to changes by ±3% in fighting, feeding, and resting compared to a fixed baseline. Recently, Siriani et al. [45] successfully used YOLO to detect laying hens in an aviary with 99.9% accuracy in low-quality videos.

The latest systems based on image analyses use neural network technology to obtain information about animal health and welfare. Regarding health, [7] used CNN algorithms as a tool to detect the emergence of gut infections based on faeces images in broiler chickens kept in multi-tier cages by Faster R-CNN and YOLOv3, with average precisions of 93.3% and 84.3%, respectively. Similarly, Mbelwa et al. [46] used CNN technology to predict broiler chicken health statuses based on images of bird droppings.

In our study, according to the validation dataset results, hens on the floor and dust-bathing hens were detected with an average precision of 61.4% and 62.9% for YOLOv4-tiny and YOLOv4, respectively. Considering the detection of hens on the floor individually, the average precisions were 94.5% and 94.1% for YOLOv4-tiny and YOLOv4, respectively. These latter performance metrics are comparable to those obtained by previous studies [45,47]. On the other hand, the average precision for the classification of dust-bathing hens was only 28.2% and 31.6% for YOLOv4-tiny and YOLOv4, respectively. Thus, the main differences found between the two versions of YOLO were related to the identification of dust bathing, since YOLOv4 was able to achieve an accuracy higher than 3.4% compared to YOLOv4-tiny. On the other hand, YOLOv4-tiny was able to classify hens on the floor and dust-bathing hens much faster than YOLOv4 (208.5 FPS vs. 31.35 FPS).

In our study, although the value of precision in the classification of dust-bathing hens was much lower than that obtained for classification of hens on the floor, this value represents the first application of YOLO for the identification of dust-bathing behaviour. In fact, dust bathing has a functional purpose in laying hens since it permits them to reset their feathers and remove excess lipids from the skin, and the process contributes to their protection from parasites [19]. However, dust bathing is an active behaviour that includes many actions around a litter area (e.g., bill raking, head rubbing, scratching with one and/or two legs, side lying, ventral lying, vertical wing shaking), as well as a synchronous behaviour (which can imply overcrowding of the litter area) [19]. Thus, both the identification of dust-bathing hens during labelling and the classification of dust-bathing hens by the ML algorithms were likely affected by the different positions that dust-bathing hens assume, thus adding a considerable level of uncertainty to our findings as the identification of the right moment when the dust bath begins is stochastic. To reduce the effects of this uncertainty, online real-time tracking could be implemented in order to consider the temporal variation and movements of hens [47]. Finally, a limited number of images (1150 images, 122 MB) was sufficient to train an object-detection algorithm with good results on hens on the floor by using an affordable and widespread platform (Google Colaboratory), thus minimizing the digital impact [48]. In fact, it is increasingly necessary to train suitable models with a limited number of images [49].

## 5. Conclusions

Under the conditions of the present study, machine learning based on the YOLO algorithm successfully identified laying hens on the floor, regardless of their activity, which could be useful for the control of the challenging piling behaviour of hens during rearing and for the implementation of on-farm early alert systems. On the other hand, while the on-farm evaluation of hens performing comfort behaviour would be useful for measuring the general welfare condition of hens or the occurrence of any challenging event or factors, the classification of dust-bathing hens was rather poor. Nevertheless, this is the first application related to such a behaviour, which comprises a well-defined sequence of different movements, for which PLF tools other than image analysis might be more successful for the classification of dust-bathing behaviours. The peculiarity of images of dust-bathing hens, in terms of illumination and geometry, can lead to complicated classification. On the other hand, the present work took advantage of a relatively low number of training images (1150), where an even further reduction would be desirable in order to ease actual field applications.

## Figures and Tables

**Table 1:** Training time, final mAP@50, and best mAP@50 (area under the precision–recall curve with a grade of overlapping bounding boxes of 50%) of the two trained models obtained on the test dataset.

Model	Training Time	Final mAP@50	Best mAP@50
YOLOv4-tiny	4.26 h	90.3%	91.7%
YOLOv4	23.2 h	87.8%	90.0%

**Table 2:** Metrics of the two models on the validation dataset (average precision for each class) for the classification of hens on the floor and dust-bathing hens.

Model	IoU	Precision	Recall	F1-score	mAP	FPS
YOLOv4-tiny	78.3%	0.94	0.93	0.92	61.4%	208.5
Hens on floor					94.5%	
Dust-bathing hens					28.2%	
YOLOv4	77.1%	0.90	0.93	0.92	62.9%	31.35
Hens on floor					94.1%	
Dust-bathing hens					31.6%	

## Data Availability

The data presented in this study are available on request from the corresponding author.
